# Tanshinone IIA-loaded nanoparticles and neural stem cell combination therapy improves gut homeostasis and recovery in a pig ischemic stroke model

**DOI:** 10.1038/s41598-023-29282-9

**Published:** 2023-02-13

**Authors:** Julie H. Jeon, Erin E. Kaiser, Elizabeth S. Waters, Xueyuan Yang, Jeferson M. Lourenco, Madison M. Fagan, Kelly M. Scheulin, Sydney E. Sneed, Soo K. Shin, Holly A. Kinder, Anil Kumar, Simon R. Platt, Jeongyoun Ahn, Kylee J. Duberstein, Michael J. Rothrock, Todd R. Callaway, Jin Xie, Franklin D. West, Hea Jin Park

**Affiliations:** 1 Department of Nutritional Sciences, University of Georgia, Athens, GA USA; 2 Department of Animal and Dairy Science, University of Georgia, Athens, GA USA; 3 Regenerative Bioscience Center, University of Georgia, Athens, GA USA; 4 Biomedical and Health Sciences Institute, University of Georgia, Athens, GA USA; 5 Environmental Health Science Department, University of Georgia, Athens, GA USA; 6 Department of Chemistry, University of Georgia, Athens, GA USA; 7 Interdisciplinary Toxicology Program, University of Georgia, Athens, GA USA; 8 Department of Small Animal Medicine and Surgery, University of Georgia, Athens, GA USA; 9 Department of Statistics, University of Georgia, Athens, GA USA; 10 US National Poultry Research Center, USDA-ARS, Athens, GA USA; 11 Present Address: Department of Molecular and Cellular Biology, Baylor College of Medicine, Houston, TX USA; 12 Present Address: Department of Industrial and Systems Engineering, Korea Advanced Institute of Science and Technology, Daejeon, South Korea

**Keywords:** Neuroscience, Stroke

## Abstract

Impaired gut homeostasis is associated with stroke often presenting with leaky gut syndrome and increased gut, brain, and systemic inflammation that further exacerbates brain damage. We previously reported that intracisternal administration of Tanshinone IIA-loaded nanoparticles (Tan IIA-NPs) and transplantation of induced pluripotent stem cell-derived neural stem cells (iNSCs) led to enhanced neuroprotective and regenerative activity and improved recovery in a pig stroke model. We hypothesized that Tan IIA-NP + iNSC combination therapy-mediated stroke recovery may also have an impact on gut inflammation and integrity in the stroke pigs. Ischemic stroke was induced, and male Yucatan pigs received PBS + PBS (Control, n = 6) or Tan IIA-NP + iNSC (Treatment, n = 6) treatment. The Tan IIA-NP + iNSC treatment reduced expression of jejunal TNF-α, TNF-α receptor1, and phosphorylated IkBα while increasing the expression of jejunal occludin, claudin1, and ZO-1 at 12 weeks post-treatment (PT). Treated pigs had higher fecal short-chain fatty acid (SCFAs) levels than their counterparts throughout the study period, and fecal SCFAs levels were negatively correlated with jejunal inflammation. Interestingly, fecal SCFAs levels were also negatively correlated with brain lesion volume and midline shift at 12 weeks PT. Collectively, the anti-inflammatory and neuroregenerative treatment resulted in increased SCFAs levels, tight junction protein expression, and decreased inflammation in the gut.

## Introduction

Ischemic stroke is a major cause of morbidity and mortality in the United States, accounting for 150,005 deaths in 2019^1^. Ischemic stroke not only directly affects the brain, but also has broad-reaching effects on the gastrointestinal (GI) system. In stroke patients, up to 50% experience GI complications, including dysphagia, constipation, and GI dysmotility, which correlate with poor recovery outcomes such as increased hospital length of stay, mortality, and poor neurological scores^2,3^. In preclinical animal model studies, ischemic stroke impairs gut motility^4^ and increases gut inflammation^5^ and mucosal damage, leading to elevated gut permeability and translocation of bacteria to the circulatory system^6,7^, as well as increased systemic inflammation^8^. These results highlight the strong relationship between the gut and the stroked brain.

The communication system between the gut and brain, the gut–brain axis, has proven to play a critical role in regulating brain function and gut homeostasis in neurological diseases such as stroke^9^. This bidirectional axis functions through neural pathways, the immune system, and the endocrine system^10,11^. The stimulated vagus nerve and activated hypothalamus–pituitary–adrenal axis interact with enteric macrophage and dendritic cells, altering gut function and integrity, leading to disruption of microbial composition^10,11^. Microbes in the gut produce neurotransmitters, including serotonin, tryptophan, γ-aminobutyric acid (GABA), and microbial metabolites such as short-chain fatty acids (SCFAs), which enter the bloodstream and interact with the host's immune system, enteric nervous system and vagus nerve to transmit information to the brain^10,11^. Previous studies have shown systemic administration of anti-inflammatory agents in rodent models of stroke not only reduced brain lesion volume and neurological deficits, but also attenuated gut-, systemic- and neuroinflammation^12,13^. Moreover, administration of healthy fecal microbiota to mice post-stroke significantly reduced infarct volume, brain edema and neurological impairment^14^. These findings highlight the dynamic interplay between the brain and gut through the gut–brain axis and the potential of this axis to be a novel therapeutic target to improve stroke outcomes.

The primary stroke injury leads to significant cell death and a secondary injury cascade of free radical formation and activation of a robust immune response which causes a cycle of increasing tissue damage in the brain^15,16^. Targeting this secondary injury cascade has been a major focus of stroke therapeutic development. Recently, our research team demonstrated that Tanshinone IIA (Tan IIA), an antioxidant and anti-inflammatory agent^17,18^, encapsulation into poly lactic-*co*-glycolic acid nanoparticles (Tan IIA-NPs) suppressed oxidative stress and inflammation^19^. Our team also demonstrated Tan IIA-NPs reduced hemispheric swelling, midline shift, and lesion volumes and improved functional deficits including changes in key spatiotemporal and kinetic gait parameters during the acute stroke stage^19^. In a separate study, our research team demonstrated that induced pluripotent stem cell-derived neural stem cells (iNSCs) transplanted into the brain acutely after stroke can replace damaged and dead neurons and glia and improve white matter integrity, cerebral blood perfusion, and brain metabolism in a pig model of ischemic stroke^20^. The combination of Tan IIA-NPs and iNSCs improved efficacy and engraftment of iNSCs, brain tissue recovery, and neurological function during the chronic stages of ischemic stroke in the porcine model^21^. However, questions remain as to how brain-targeted stroke therapies alter the GI system and potentially inhibit GI induced systemic and secondary inflammation in the stroke brain.

In the present study, we aimed to investigate how ischemic stroke and treatment of Tan IIA-NP + iNSC combination therapy delivered to the brain alters gut homeostasis and gut integrity in a porcine ischemic stroke model. The pig is a robust translational animal model with similar brain and GI anatomy and physiology to humans, making pigs an ideal model for future clinical studies^22–25^. The findings from the translational pig model will provide a better understanding of the bidirectional communication between the brain and gut and inform future efforts to develop stroke therapeutics.

## Results

### Tan IIA-NP + iNSC treatment reduced inflammation and improved expression of tight junction proteins in jejunal mucosa 12 weeks post-treatment

**Figure 1:**
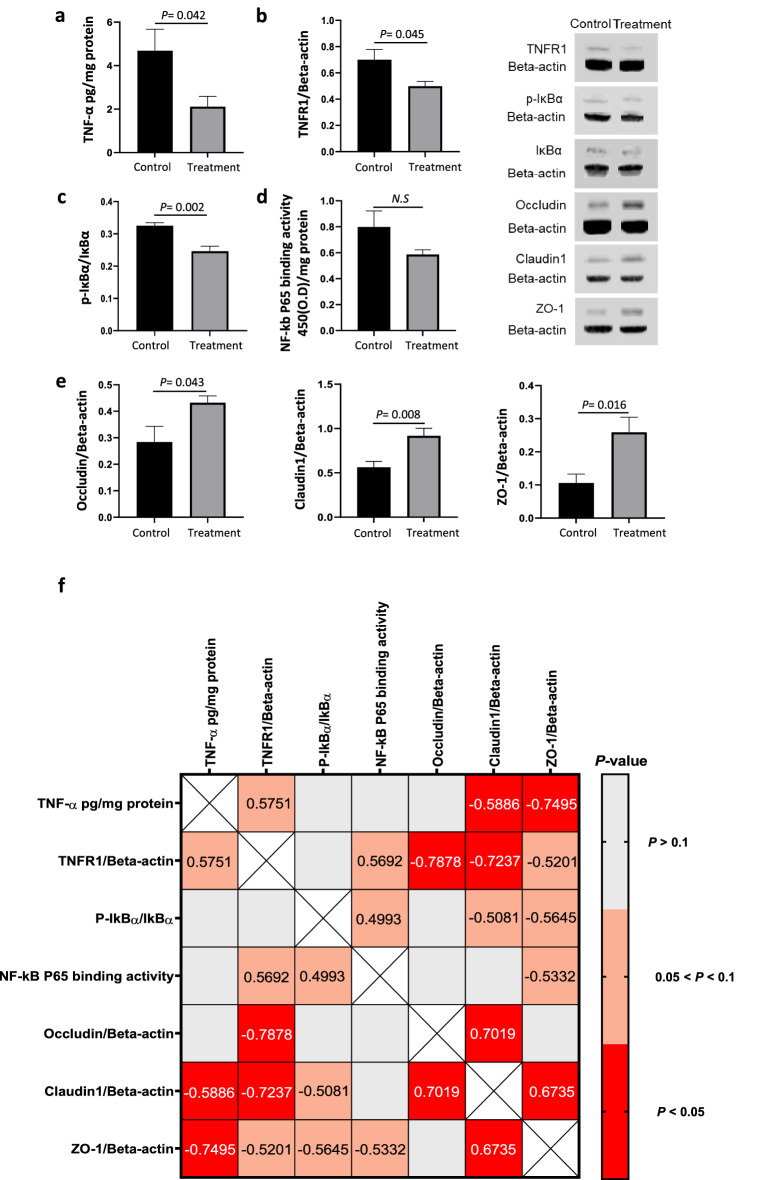
Tan IIA-NP + iNSC treatment reduced inflammation and improved expression of tight junction proteins in jejunal mucosa at 12 weeks post-treatment. At 12 weeks PT, treated pigs showed decreased protein levels of (**a**) TNF-α, (**b**) TNFR1, (**c**) phosphorylation of IkBα relative to the control. (**d**) Treatment decreased binding activity of NF-kB P65 compared to control although not statistically significant. (**e**) Treatment significantly increased protein expression of gut tight junction proteins occludin, claudin1, and ZO-1 compared to control. Unpaired t-test N = 6 each group. (**f**) Protein levels of TNF-α, TNFR1, phosphorylated IkBα, and binding activity of NF-kB P65 were correlated with the expression of gut tight junction proteins occludin, claudin1, and ZO-1. Significant correlations (*P* value < 0.05) are highlighted in red while trending correlations are represented in orange. The number inside each box represents the r-value, r = Pearson correlation coefficient.

To evaluate the effect of the Tan IIA-NP + iNSC combination therapy on gut inflammation, protein expression of TNF-α, TNF-α receptor1 (TNFR1), and binding activity of NF-kB P65 were examined in jejunal scrapings at 12 weeks PT (Fig. 1). The Tan IIA-NP + iNSC group showed reduced protein levels of proinflammatory cytokine TNF-α by 54.91% (Control 4.68 ± 0.99 vs. Treatment 2.11 ± 0.48, *P* = 0.042, Fig. 1a) and its receptor TNFR1 by 28.57% (Control 0.70 ± 0.08 vs. Treatment 0.50 ± 0.04, *P* = 0.045, Fig. 1b) compared to the control group. NF-kB plays an important role in regulating the inflammatory response and it induces inflammation through IkBα phosphorylation, an NF-kB inhibitor, and activation of transcriptional factors such as P65^26^. Interestingly, Tan IIA-NP + iNSC treatment also reduced protein levels of phosphorylated IkBα (Control 0.33 ± 0.01 vs. Treatment 0.25 ± 0.02, *P* = 0.002, Fig. 1c) compared to the control group. Binding activity of NF-kB P65 showed a decreasing trend in the treated group relative to the control group (Control 0.80 ± 0.12 vs. Treatment 0.56 ± 0.04, 0.1 > *P* > 0.05, Fig. 1d). These results suggest that Tan IIA-NP + iNSC therapy reduces the levels of proinflammatory cytokines and activation of NF-kB signaling in the pig model of ischemic stroke.

To investigate the effect of the Tan IIA-NP + iNSC combination therapy on gut integrity, protein expressions of gut tight junction proteins occludin, claudin1, and ZO-1 were measured in jejunal mucosa at 12 weeks PT (Fig. 1e). The results showed that Tan IIA-NP + iNSC treated animals had 1.5-fold higher protein expression of occludin (Control 0.28 ± 0.06 vs. Treatment 0.43 ± 0.03, *P* = 0.043), a 1.6-fold higher expression level of claudin 1 (0.56 ± 0.07 vs. 0.92 ± 0.08, *P* = 0.008), and a 2.4-fold higher expression level of ZO-1 (0.11 ± 0.03 vs. 0.26 ± 0.05, *P* = 0.016) compared to the control group. These results indicate that the Tan IIA-NP + iNSC treatment preserves gut tight junction protein expression following stroke.

### Gut inflammation is negatively correlated with gut tight junction protein expression at 12 weeks post-treatment

To further evaluate the association between the gut inflammatory response and gut integrity, correlations were assessed between proinflammatory cytokines TNF-α and TNFR1 and gut tight junction proteins occludin, claudin1, and ZO-1 12 weeks PT in jejunal mucosa samples (Fig. 1f). Increased protein levels of TNF-α (claudin1: r = − 0.5886, *P* = 0.044, ZO-1: r = − 0.7495, *P* = 0.005) and TNFR1 (occludin: r = − 0.7878, *P* = 0.002, claudin1: r = − 0.7237, *P* = 0.008, ZO-1: r = − 0.5201, *P* = 0.083) negatively correlated with gut tight junction protein expression, thus signifying the detrimental effects of TNF-α on gut integrity. Increased protein levels of phosphorylated IkBα (p-IkBα/total IkBα) (claudin1: r = − 0.5081, *P* = 0.092, ZO-1: r = − 0.5645, *P* = 0.056) and NF-kB P65 binding activity (ZO-1: r = − 0.5332, *P* = 0.074) showed a trending negative correlation with tight junction protein expression, although not statistically significant. Interestingly, protein levels of TNF-α were positively correlated with TNFR1 (r = 0.5751, *P* = 0.050), and high TNFR1 levels were associated with elevated NF-kB P65 binding activity (r = 0.5692, *P* = 0.053). NF-kB P65 binding activity had a trending positive correlation with phosphorylation of IkBα (r = 0.4993, *P* = 0.098), indicating an association between the TNF-α, TNFR1, and NF-kB signaling. Collectively, these results suggest that the decrease in tight junction protein expression in gut epithelial cells following stroke may be mediated by the proinflammatory cytokine TNF-α and NF-kb pathway in ischemic stroke.

### Tan IIA-NP + iNSC therapy altered fecal SCFAs levels in an ischemic stroke pig model

**Figure 2:**
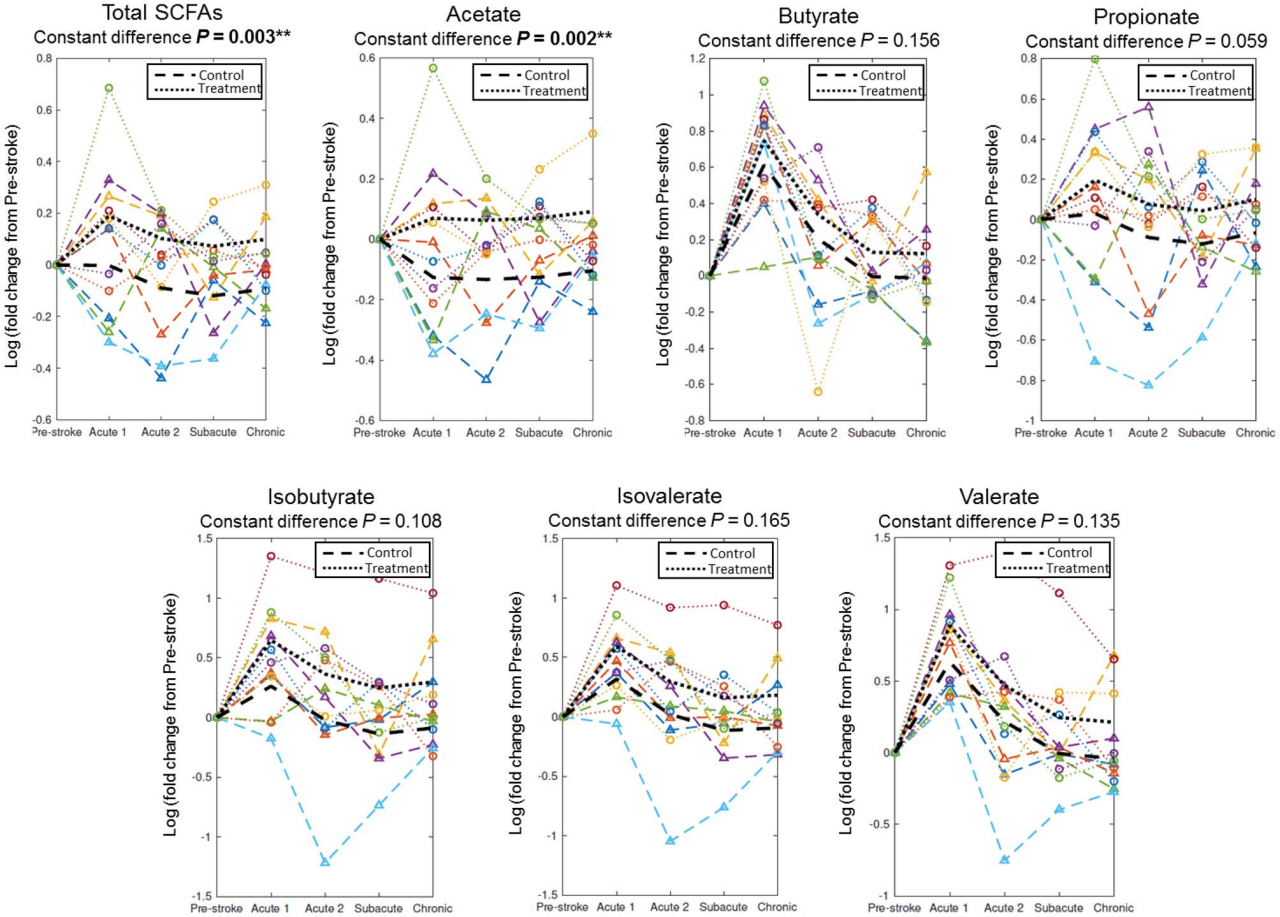
Tan IIA-NP + iNSCs combination therapy increased fecal SCFAs levels in ischemic stroke pigs. Longitudinal changes in fecal SCFAs levels were examined by a multivariate quadratic regression model. Colored lines represent the individual changes in fecal SCFAs of pigs used in this study. The two black lines represent the changes in fecal SCFAs in the control group (n = 6) and the treatment group (n = 6). Changes in fecal total SCFAs and acetate levels from baseline were significantly higher in the treatment group compared to the control group PS. **P < 0.01.

Longitudinal changes in fecal SCFAs levels were analyzed pre-stroke and up to 12 weeks PT. Changes in fecal SCFAs levels from baseline (pre-stroke values) at each timepoint of stroke including Acute 1 (1–5 days post-stroke), Acute 2 (1–3 days PT), Subacute (1–4 weeks PT), and Chronic (6–12 weeks PT) were calculated and shown in Fig. 2. There were significant differences in SCFAs between Tan IIA-NP + iNSC treated and control animals with the Tan IIA-NP + iNSC treated animals consistently demonstrating higher levels of SCFAs relative to controls (Fig. 2). Particularly, the levels of fecal total SCFAs (*P* = 0.003) and acetate (*P* = 0.002) were significantly higher in the treatment group compared to the control group up to 12 weeks PT. The changes in fecal propionate levels (*P* = 0.059) showed trending higher levels in the treatment group than in the control group, whereas the changes in fecal butyrate, isobutyrate, isovalerate, and valerate levels had no significant differences between groups PS (*P* > 0.05). These results suggest Tan IIA-NP + iNSC combination therapy leads to increased intestinal levels of SCFAs during PS recovery.

### Changes in fecal SCFAs levels during stroke recovery correlated with jejunal inflammation and ZO-1 expression 12 weeks post-treatment

**Figure 3:**
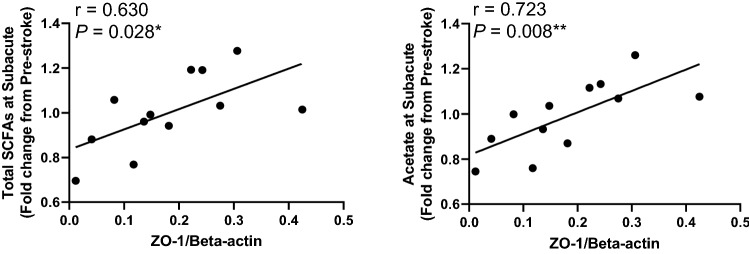
Total SCFAs and acetate levels at the subacute stage were positively correlated with ZO-1 protein expression in the jejunal mucosa at 12 weeks post-treatment. Fecal total SCFAs and acetate levels at the subacute was positively correlated with ZO-1 protein expression at 12 weeks PT (All *P* < 0.05). r = Pearson correlation coefficient and *P* values are shown. *P < 0.05, **P < 0.01.

To assess the association between the gut inflammatory response and gut integrity with SCFAs levels, protein levels of TNF-α, TNFR1, phosphorylation of IkBα, gut tight junction proteins, and binding activity of NF-kB P65 at 12 weeks PT were correlated with changes in fecal SCFAs levels post-stroke. Changes in fecal total SCFAs and acetate levels at Subacute were negatively correlated with the protein levels of TNF-α and phosphorylated IkBα (Table 1), and positively correlated with the expression of gut tight junction protein ZO-1 at 12 weeks PT (Fig. 3). Changes in fecal isobutyrate and isovalerate levels at Acute 2 and Subacute stages were also negatively correlated with the protein levels of phosphorylated IkBα at 12 weeks PT (Table 1). These results indicate that increased SCFAs levels in the gut at early stages PT (1 day to 4 weeks PT) are related to reduced gut inflammation and improved gut integrity in later stroke stages. Interestingly, changes in fecal butyrate levels at the Chronic stage had a significant positive correlation with protein levels of TNF-α, TNFR1, and NF-kB P65 binding activity at 12 weeks PT. Collectively, changes in SCFAs levels in the gut following stroke may be related to levels of gut inflammation and membrane permeability over an extended period.

**Table 1:** Correlations between changes in fecal SCFAs levels post-stroke and jejunal inflammation at 12 weeks post-treatment in a pig model of ischemic stroke.

Post-stroke	12 weeks PT			
	TNF-α	TNF-α receptor 1	Ikbα phosphorylation	NF-kB P65 binding activity
Total SCFAs	*P* = 0.032^c^	N.S	*P* = 0.041^c^	N.S
	r = − 0.619		r = − 0.595	
Acetate	*P* = 0.031^c^	N.S	*P* = 0.031^c^	*P* = 0.029^c^
	r = − 0.622		r = − 0.623	r = − 0.628
Butyrate	*P* = 0.030^d^	*P* = 0.031^d^	N.S	*P* = 0.014^d^
	r = 0.624	r = 0.622		r = 0.075
Propionate	N.S	N.S	N.S	N.S
Isobutyrate	N.S	N.S	*P* = 0.031^b^, 0.011^c^	N.S
			r = − 0.623, − 0.700	
Isovalerate	N.S	N.S	*P* = 0.039^b^, 0.009^c^	N.S
			r = − 0.600, − 0.716	
Valerate	N.S	N.S	*P* = 0.024^b^	N.S
			r = − 0.644	

Time point of fecal SCFAs showing significant correlation: a = Acute 1, b = Acute 2, c = Subacute, d = Chronic. r = Pearson correlation coefficient.

### The jejunal protein levels of phosphorylated IkBα correlated with lesion volume and midline shift changes 12 weeks post-treatment

**Figure 4:**
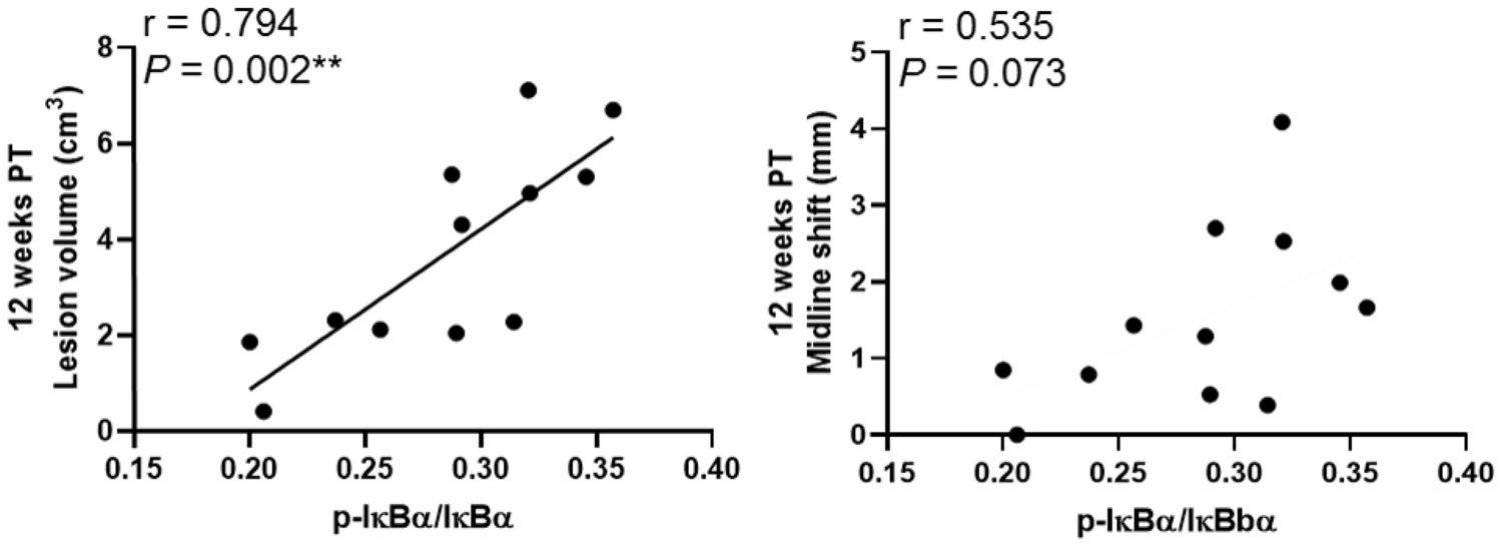
Protein levels of phosphorylated IkBα correlated with the brain lesion volume and midline shift changes 12 weeks post-treatment. Lesion volume and midline shift at 12 weeks PT were positively correlated with jejunal protein levels of phosphorylated IkBα at 12 weeks PT. r = Pearson correlation coefficient and *P* values are shown. *P < 0.05, **P < 0.01.

To investigate the association between the gut inflammatory response and stroke brain volumetric changes, protein levels of TNF-α, TNFR1, phosphorylated IkBα, gut tight junction proteins, and binding activity of NF-kB P65 at 12 weeks PT were correlated with MRI lesion volume and midline shift results at 12 weeks PT (Fig. 4). Lesion volume (r = 0.794, *P* = 0.002) and midline shift (r = 0.535, *P* = 0.073) were positively correlated with the protein levels of phosphorylated IkBα in the gut at 12 weeks PT (Fig. 4). This result suggests that elevated gut inflammation may be associated with cerebral tissue damage.

### Changes in fecal SCFAs levels post-stroke correlated with lesion volume and midline shift changes 12 weeks post-treatment

Previously, our research team reported that intracisternal administration of Tan IIA-NPs and iNSCs transplantation therapy reduced brain tissue damage 12 weeks PT compared to the non-treated control group^21^. In this study, we correlated changes in gut SCFAs levels with stroke-induced tissue damage at 12 weeks PT (Table 2). The changes in fecal total SCFAs, acetate, isobutyrate, isovalerate, and valerate levels at Acute 2 had a negative correlation with lesion volume at 12 weeks PT (Table 2). The changes in fecal total SCFAs, acetate, isobutyrate, and isovalerate levels at Acute 2 also had an inverse correlation with midline shift at 12 weeks PT. Collectively, these results show that changes in fecal SCFAs levels post-stroke may affect cerebral tissue damage, associated with stroke recovery.

**Table 2:** Correlations between post-stroke changes in fecal SCFAs levels and lesion volume and midline shift changes 12 weeks post-treatment.

Post-stroke	12 weeks PT	
	Lesion volume (cm^3^)	Midline shift (mm)
Total SCFAs	*P* = 0.027^b^	*P* = 0.037^b^
	r = − 0.634	r = − 0.605
Acetate	*P* = 0.038^b^	*P* = 0.037^b^
	r = − 0.602	r = − 0.604
Butyrate	N.S	N.S
Propionate	N.S	N.S
Isobutyrate	*P* = 0.006^b^	*P* = 0.042^a^, 0.012^b^
	r = − 0.741	r = − 0.593, − 0.694
Isovalerate	*P* = 0.004^b^, 0.038^c^	*P* = 0.007^b^
	r = − 0.763, − 0.604	r = − 0.730
Valerate	*P* = 0.020^b^	*P* = 0.039^a^
	r = − 0.659	r = − 0.600

Time point of fecal SCFAs showing significant correlation: a = Acute 1, b = Acute 2, c = Subacute, d = Chronic. r = Pearson correlation coefficient.

## Discussion

This study demonstrated that the Tan IIA-NP + iNSC combination therapy modulated gut homeostasis in a MCAO ischemic stroke pig model as indicated by reduced proinflammatory cytokine levels, improved tight junction protein expression in the jejunum, and increased fecal SCFAs levels. Moreover, higher fecal SCFAs levels PS were associated with decreased brain lesion volume and midline shift. The findings from this study suggest that the antioxidative, anti-inflammatory, and neuroregenerative Tan IIA-NP + iNSC treatment resulted in an improved gut inflammatory status and integrity via brain–gut axis interactions in a pig model of ischemic stroke.

The proinflammatory immune response is a key contributor to stroke sequelae and resulting pathology^27^. During stroke, damaged and dying neural cells release damaged-associated molecular patterns (DAMPs) that activate microglia and astrocytes at the lesion site and enhance recruitment and parenchymal invasion of leukocytes from peripheral circulation^28^. Recently, our research team demonstrated that treatment with Tan IIA-NP + iNSC reduced the number of microglial and infiltrating immune cells in the brain, maintained endogenous neurons, and enhanced populations of endogenous neural stem cells in pigs^21^. Activated microglia and macrophages release proinflammatory cytokines such as TNF-α and IL-1β in the brain^29^. The released DAMPs and these cytokines can enter systemic circulation through the impaired blood–brain barrier and the cerebrospinal fluid drainage system^30^. Therefore, the anti-inflammatory effect of Tan IIA-NP + iNSC on the cerebral immune response may also mitigate systemic and gut inflammation. This is supported by the present study as the Tan IIA-NP + iNSC treatment led to reduced protein levels of proinflammatory cytokines (e.g., TNF-α) and improved tight junction protein expression levels in the gut.

Inflammatory processes are closely related to the GI dysfunction, such as compromised gut permeability^31^. Increased gut permeability can allow harmful antigens and microorganisms to enter systemic circulation from the intestinal lumen, resulting in abnormal immune reactions. Therefore, changes in intestinal permeability are important indicators of gut health^32^. Previous studies showed that administration of proinflammatory cytokines IL-1β and TNF-α to Caco-2 cells in vitro increased epithelial permeability in a dose- and time-dependent manner^33,34^. Therefore, these results suggest proinflammatory cytokines play a crucial role in regulating intestinal barrier function. Interestingly, the present study reported that treatment with Tan IIA-NP + iNSC reduced TNF-α and TNFR1 protein levels and increased occludin, claudin1, and ZO-1 protein expression in the gut. It has been suggested that TNF-α mediates the expression of tight junction proteins through a specific molecular mechanism^35^. Briefly, TNF-α binds to the TNF-α receptor in the epithelial cells of the gut and stimulates the NF-kB signaling pathway by increasing myosin light chain kinase (MLCK) gene expression^35,36^. MLCK then phosphorylates myosin light chain (MLC) and phosphorylated MLC induces contraction of the cytoskeletal structure attached to the tight junction proteins, increasing the gut permeability^35–37^. Comparatively, our study reported that protein levels of jejunal TNF-α were positively correlated with the protein TNFR1, both of which were negatively correlated with gut tight junction protein expression. Therefore, the findings from this study indicate that intestinal TNF-α levels are an important factor in regulating tight junction-mediated gut permeability in pigs.

The transcriptional factor NF-kB is a critical mediator of inflammatory stimuli^26^. NF-kB is a family of inducible transcription factors such as NF-B1 (p50), NF-B2 (p52), and RelA (p65). This family binds to specific regions of DNA elements within enhancers or promoters of target genes and influences gene expression^38^. In general, NF-kB proteins are sequestered in the cytoplasm by IkB family, an inhibitory proteins; however, when the NF-kB pathway is activated and IkB is phosphorylated, the NF-KB transcriptional factors are released and enter the nucleus to promote gene expression^39^. The present study showed that the intestinal binding activity of NF-kB P65 was lower in Tan IIA-NP + iNSC treated animals than in non-treated animals, although it was not statistically significant. The NF-kB P65 binding activity had a trending positive correlation with the protein levels of phosphorylated IkBα, suggesting the involvement of NF-kb pathway in this study. As mentioned in the paragraph above, the activated NF-kb pathway can be induced by proinflammatory cytokines such as TNF-α and elevate MLCK gene expression, thereby reducing the expression of gut tight junction proteins in epithelial cells^40,41^. Similarly, the present study found that the binding activity of NF-kB P65 also tended to positively correlate with the protein levels of TNFR1 and negatively correlate with ZO-1 in the gut. These results propose that TNF-α induced NF-kB signaling may be an integral pathway for modulating gut integrity in this ischemic stroke pig model.

SCFAs are major metabolites produced by the gut microbiota when undigested carbohydrates are fermented and mainly consist of acetate, propionate, and butyrate. In the intestinal tract, SCFAs regulate gut epithelial barrier function, integrity, and immune response^42^, and also play a crucial role as mediators in gut–brain interaction^10,11^. The present study showed that stroked pigs that received Tan IIA-NP and iNSC therapies had increased changes in fecal SCFAs levels throughout the study period and these changes not only correlated with reduced gut inflammation and improved gut integrity, but also were associated with reduced lesion volumes and midline shift 12 weeks PT. SCFAs can be absorbed by colonocytes and interact with intestinal immune cells, as well as cross the blood–brain barrier through the circulatory system and modulate astrocyte activation and microglia maturation in the brain^43–45^. Previous studies reported that increased SCFAs levels in the gut, brain, and plasma through fecal transplantation reduced PS neurological deficit and neuroinflammation, suggesting that intestinal SCFAs may affect the brain through blood circulation^46^. Moreover, SCFAs supplementation in drinking water improved PS recovery by increasing synaptic plasticity and cortical reorganization and reducing microglial activation and frequency of T-cells in the brain of a mouse model of ischemic stroke^47^, indicating a significant effect of SCFAs on recovery mechanisms in the brain. Although the current study did not measure the plasma levels of SCFAs, these previous studies suggest that changes in SCFAs levels following Tan IIA-NP + iNSC treatment may be associated with improved stroke pathological recovery.

Gut dysbiosis in ischemic stroke results in dysregulated gut–brain signaling and the restoration of the gut microbiome improves stroke outcomes by regulating metabolic, immune, and inflammatory responses^48^. The inflamed gut environment in ischemic stroke can alter SCFAs levels due to the overgrowth of pathogenic bacteria such as Enterobacteriaceae or *Escherichia coli*, and hinder the growth of beneficial bacteria such as commensal and SCFAs-producing bacteria^49–52^. In the current study, the inflamed gut environment may be improved following the treatment due to the anti-inflammatory and antioxidant effect of Tan IIA^17,19^ and iNSC^20^ treatment. The reduced cytotoxic stress in the stroke brain following the anti-inflammatory and antioxidant Tan IIA and iNSC treatment is likely to decrease the systemic immune response and further influence the gut environment by reducing the production of reactive oxygen species. This is likely to lead to the inhibition of the overgrowth of harmful bacteria and to the growth of SCFAs-producing bacteria resulting in the observed increase in fecal SCFAs levels.

The most abundant SCFAs in all groups are acetate, propionate, and butyrate, and have been widely investigated on their effects on host health^42^. Branched short-chain fatty acids (BCFAs), such as isobutyrate and isovalerate are produced in lower amounts by gut microbes that ferment branched-chain amino acids^53^ and the impact of BCFAs on host health is largely unknown. Interestingly, the present study found that changes in fecal levels of isobutyrate and isovalerate during the acute stage of stroke were negatively correlated with lesion volume and midline shift changes in the chronic stroke stage. Similarly, Zhang and Chen reported that rodent models of stroke showed increased fecal isobutyrate and isovalerate levels in the early stage of stroke^12,14^, while displaying decreased fecal levels of butyrate^12^ or acetate and propionate^14^ PS. Increased BCFAs levels in the early stage of stroke may be due to a compensation mechanism after the depletion of the major SCFAs caused by gut dysbiosis. Therefore, these findings suggest that BCFAs may play an important role in regulating stroke recovery. However, given the lack of studies evaluating BCFAs changes and their effect on stroke, further studies are needed to investigate the potential beneficial role of BCFAs in human health.

In this study, the combination of Tan IIA-NPs and iNSCs was used as a treatment. In the same pig cohort, our team found that the Tan IIA-NP + iNSC treatment had smaller lesion volume and midline shift 1 day post-stroke and 12 weeks post-treatment compared to the control and the iNSC only treated group^21^. This MRI result at 1 day post-stroke indicates that Tan IIA-NP alone have a neuroprotective effect on stroke injury because iNSCs were not transplanted until 5 days post-stroke. Tan IIA is loaded into PLGA nanoparticles, which are biodegradable nanoparticle that have controlled release properties to slowly release Tan IIA over time. Tan IIA was found to reach approximately 60% release at 50 h^19^. Therefore, the MRI results found 1 day post-stroke in Tan IIA-NP + iNSC group suggests an acute therapeutic effect of Tan II-NPs on stroke severity. iNSCs are a promising stroke treatment due to their replacement and regenerative properties that help neural recovery^54^. However, iNSCs have a short post-transplantation survival time because of the significant inflammatory response and the build up of free radicals in stroked tissue. This occurs within a very short time window (mins to hours) post-stroke^55^, and Tan II-NP can mitigate this secondary injury having anti-inflammatory and antioxidative properties. Therefore, rather than examining the effects of Tan IIA-NP on its own, we sought to look at the effect of Tan IIA-NPs combined with iNSC, which is more relevant to clinical application.

## Conclusion

The findings of this study demonstrate that intracisternal Tan IIA-NPs and intracerebral iNSC therapies decreased jejunal inflammation and membrane permeability and increased fecal SCFAs levels in the gut. This seminal study suggests that neuroprotective and regenerative stroke therapies enhance gut homeostasis, potentially through gut-brain interaction, and decreased stroke pathology.

## Methods

### Animal information

All experiments were performed in accordance with the National Institutes of Health Guide for the Care and Use of Laboratory Animals guidelines and were approved by the University of Georgia (UGA) Institutional Animal Care and Use Committee (IACUC; Protocol Number: 2017–07–019Y1A0). The present study is reported in accordance with ARRIVE guidelines. Castrated male Yucatan pigs (9-month-old, 31–41 kg body weight) were enrolled in this study. Pigs were individually housed in a 12-h light–dark cycle with a room temperature of approximately 27 °C. All pigs had free access to water and were fed standard grower diets.

### Treatment and group information

**Figure 5:**
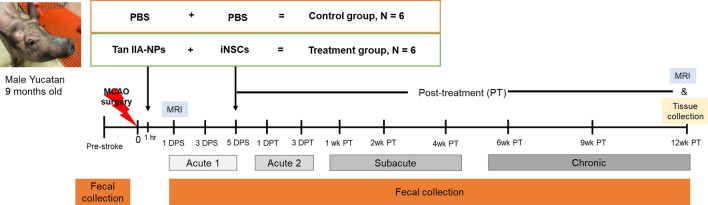
Experimental study design. Castrated male Yucatan pigs (9-month-old, 31–41 kg body weight) underwent middle cerebral artery occlusion (MCAO) and received either PBS (Control group, n = 6) or Tan IIA-NPs and iNSCs (Treatment group, n = 6). Either PBS or Tan IIA-NPs were administered intracisternally at 1 h post-stroke (PS) and PBS or iNSCs were transplanted into the perilesional region at 5 days PS. Ischemic stroke was confirmed at 1 day PS by magnetic resonance imaging (MRI) and lesion volume and midline shift were measured 12 weeks (wk) post-transplantation (PT). PS stages were categorized into Acute 1 (1–5 days PS), Acute 2 (1–3 days PT), Subacute (1–4 week PT), and Chronic (6–12 week PT)^57,58^. Fecal samples were collected pre-stroke and at multiple timepoints PS to measure SCFAs levels. All pigs were sacrificed at 12 wk PT and distal jejunal samples were scraped and collected for further analysis.

All procedures were conducted in accordance with the ARRIVE guidelines and the experimental study design is summarized in Fig. 5. Pigs underwent middle cerebral artery occlusion surgery (MCAO) and received either PBS (Control group n = 6) or Tan IIA loaded poly(lactide-*co*-glycolide) (PLGA) nanoparticles (Tan IIA-NPs) and iNSCs treatment (Treatment group, n = 6). PLGA nanoparticles (NPs) were produced via a nanoprecipitation method^19^. iNSCs (HIP™ hNSC BC1, GlobalStem®, Rockville, MD) were purchased and cultured in neural stem cell media^20^. At 1 h post-stroke, PBS or 133 µg/kg Tan IIA-NPs were injected into the midline of the dorsal neck through the cerebrospinal fluid at the anatomical junction created by the vertical line on the rostral side of the first vertebral body wing and the horizontal line joining the dorsal arch. Five days post-stroke, PBS or 80 ul of 1.2 × 10^7^ iNSCs suspended in PBS were injected by quintessential stereotaxic injector (Stoelting) within the perilesional region to the center of the white matter. Tan IIA-NPs were administered prior to iNSC transplantation to help mitigate the cytotoxic microenvironment following stroke, thus potentially increasing the survivability and therapeutic efficacy of iNSCs. One animal in the PBS group and three animals in the Tan IIA-NP + iNSC group died post-treatment. A total of 6 animals per group that survived to the end of the experimental period were analyzed as part of this study. A more detailed method of Tan IIA-NPs injection and iNSCs transplantation can be found in Kaiser et al^21^.

### Preoperative, perioperative, and post-operative procedures

1 day prior to surgery, Excede [5 mg/kg, intramuscular (IM)] and fentanyl patches [100 mg/kg/h, transdermal (TD)] were provided to pigs as antibiotics and analgesia, respectively. On the day of surgery, analgesia and sedation were administered using xylazine (2 mg/kg, IM) and midazolam (0.2 mg/kg, IM). Propofol [10 mg/ml, intravenous (IV)] and prophylactic lidocaine (2%, topically to the laryngeal folds) were utilized to aid in endotracheal intubation. Lactated Ringer's solution (5 ml/kg/h, IV) was given to maintain hydration. Anesthesia was maintained with isoflurane (1.0–2.0%) in oxygen and air with vitals including temperature, respiration, heart rate, and blood pressure continuously monitored and maintained within normal parameters. Artificial ventilation was maintained at 8–12 breaths per minute with a tidal volume of 5–10 ml/kg. Heart rate was monitored by Doppler probe placement on the femoral artery while blood pressure was monitored by a sphygmomanometer. Rectal temperature was recorded every 15 min using a digital thermometer. MCAO surgery was performed as described previously^56^. Briefly, a curvilinear incision was made from the superior right orbit to the rostral side of the auricle. The temporalis muscle was retracted and the exposed local dura mater was excised. Using bipolar electrocautery forceps, the middle cerebral artery at the distal part of the Circle of Willis was permanently occluded. Post-surgery, pigs were recovered in intensive care unit (ICU) pens, and once extubated, were returned to their home pens. Pigs were closely monitored every 15 min until heart rate, respiration, and temperature returned to normal. Pigs were then monitored every 4 h for 24 h and twice a day thereafter. Flunixin meglumine (2.2 mg/kg, IM) was given every 12 h for the first 24 h and every 24 h for the next 3 days for post-operative pain and fever control.

### Magnetic resonance imaging

MRI was conducted (General Electric 3.0 Tesla MRI system) 1 day PS to confirm the presence of ischemic stroke and again at 12 weeks PT (Data not shown). Pigs had been sedated and sustained under anesthesia as previously mentioned for MCA occlusion operations. The cranial MRI was performed on pigs in a supine position using an 8 channel torso coil. Lesion volume and midline shift were measured at 12 weeks PT as an indicator of stroke recovery. Multiplanar MRI sequences including T2 Fluid Attenuated Inversion Recovery (T2FLAIR), Diffusion Weighted Imaging (DWI), T2Weighted (T2W), and T2Star (T2*) had been obtained and each sequence was analyzed by using the Osirix software (Version 5.6). T2FLAIR, DWI, and ADC maps were used collectively to validate the existence of ischemic stroke 1 day PS and followed up at 12 weeks PT. Lesion volume (cm^3^, T2W sequences) was identified by heyperintense regions of interest (ROIs) while midline shift (mm, T2W sequence) was calculated by measuring the distance between the exact midpoint of the length of the septum pellucidum and the ideal midline. The MRI results of the same cohort of pigs have been recently published by Kaiser et al.^21^.

### Fecal collection

Fecal samples were collected at pre-stroke, 1, 3, 5 days post-stroke (Acute 1), 1, 3 days post-treatment (Acute 2), 1, 2, 4 weeks post-treatment (Subacute), and 6, 9, 12 weeks post-treatment (Chronic). Each stroke stage was designated based on previously identified and classified human stroke stages^57,58^. To avoid any contamination during fecal collection, all materials were sterilized before sample collection with 70% alcohol. A sterilized fecal loop was introduced in the rectum and feces were collected into a 50 ml tube without touching the floor or body. After aliquoting the feces into three 2 ml tubes, they were immediately frozen on dry ice and stored at − 80 °C until further analysis.

### Short-chain fatty acids (SCFAs) analysis

Fecal sample SCFAs were analyzed as previously described^59^. The samples were suspended in water (1 g of feces in 3 mL water) and mixed using a vortex. The fecal suspension was centrifuged at 10,000*g* for 10 min and 1 mL of supernatant was pipetted into a new centrifuge tube containing 200 µL of a metaphosphoric acid solution (25% wt/vol) before freezing overnight. Samples were then thawed and centrifuged at 10,000*g* for 10 min. The supernatant was transferred to a polypropylene tube and mixed with ethyl acetate (2:1 = ethyl acetate:supernatant). The tubes were vortexed for 15 s before settling for 5 min. Afterwards, 500 µL of the upper suspension was transferred to screw-thread vials for SCFAs analysis in a Shimadzu GC-2010 Plus gas chromatograph (Shimadzu Corporation, Kyoto, Japan) with a flame ionization detector and a capillary column (Zebron ZB-FFAP; 30 m × 0.32 mm × 0.25 μm; Phenomenex Inc., Torrance, CA, USA). The injection volume was 1.0 μL and helium was used as the carrier gas. The column temperature was initially set at 110 °C and raised to 200 °C over the course of 6 min. The injector and detector temperatures were set at 250 °C and 350 °C, respectively.

### Tissue collection

At 12 weeks PT, distal jejunum (about 200 cm before the end of the ileum) tissues were collected and scraped using glass slides. The samples were immediately frozen in liquid nitrogen and then stored at − 80 °C until further analysis.

### Enzyme-linked immunosorbent assay (ELISA) assay

The proinflammatory cytokine tumor necrosis factor-alpha (TNF-α) was quantified by Porcine Quantikine ELISA (R&D company, Minneapolis, MN, USA, Cat. PTA00) in jejunal homogenate samples according to the manufacturer’s instructions. These results were normalized by the total protein concentration in each sample.

### Western blot analysis

The distal jejunum tissue was collected and scraped with a glass slide. 50–100 mg of scraped jejunal mucosa was added to 500 µL of Radioimmunoprecipitation Assay (RIPA) lysis buffer (Santa Cruz Biotechnology, CA, USA) and homogenized. Samples were then centrifuged twice at 10,000*g* for 20 min at 4 °C to collect the clear supernatant. The protein concentration was determined using the bicinchoninic acid (BCA) protein assay (Thermo Fisher, Rockford, IL, USA) following the manufacturer’s protocol. 25 µg protein was loaded on either 10% or 16% Tris–glycine gel (Invitrogen, Carlsbad, CA, USA) and transferred to nitrocellulose membrane (Invitrogen, Carlsbad, CA, USA). Membranes were blocked in either LICOR blocking buffer (LI-COR, Lincoln, NE, USA) or 5% non-fat skim milk for 1 h at room temperature prior to the addition of primary antibodies: Occludin (1:1000, Abcam, ab31721), Claudin1 (1:1000, Abcam, ab15098 ), Zonula occludens-1 (ZO-1) (1:1000, Abcam, ab214228), TNF-α receptor 1 (TNFR1) (1:1000, Abcam, ab19139), IkBα (1:1000, Cell signaling, #4814), p-IkBα (1:200, Cell signaling, #9246), and beta-actin (1:10,000, Sigma Aldrich, #A5441-100UL) overnight at 4 °C. After washing the membrane with tris-buffered saline (TBS) with tween T20 (TBS-T) buffer 4 times for 5 min, the secondary antibody, IRDye 800CW goat anti-rabbit IgG (1:10,000, LI-COR, #926-32211) and IRDye 680RD goat anti-mouse IgG (1:10,000, LI-COR, #926-68070) were incubated at room temperature for 1 h. Then, the membrane was washed with TBS-T 4 times for 5 min and stored in PBS until imaging. The membrane was imaged using the Odyssey imaging system (LI-COR, Lincoln, NE, USA) and the images were analyzed by ImageJ software (v8.4.3, San Diego, CA, USA). Beta-actin was used as a housekeeping gene and the relative abundance of target proteins to beta-actin was shown.

### NF-kB P65 binding activity assay

Nuclear protein extraction was performed in jejunum tissue using a Nuclear Extract Kit (Active Motif, Carlsbad, CA) and NF-kB P65 binding activity was measured using TransAM™ NF-kB P65 transcription factor assay kit (Active Motif, Carlsbad, CA) according to the manufacturer’s instructions.

### Statistical analysis

Longitudinal changes in fecal SCFAs levels were evaluated with a multivariable quadratic regression model to investigate the differences between the groups (MATLAB). Western blot, ELISA, and NF-kB P65 binding activity data were analyzed by GraphPad Prism software (v8.4.3, San Diego, CA, USA) using an unpaired t-test between the control and the treatment group. Data are presented as mean ± standard error of the mean (S.E.M). Pearson correlations were performed to evaluate associations between fecal SCFAs level, gut inflammation, expressions of gut tight junction protein, and cerebral tissue changes.

## Supplementary Information

Supplementary Figures.

## Data Availability

The datasets generated during and/or analyzed during the current study are available from the corresponding author on reasonable request.
