# Supplementary material for: Tanshinone IIA-loaded nanoparticles and neural stem cell combination therapy improves gut homeostasis and recovery in a pig ischemic stroke model
Source: Sci Rep. 2023 Feb 13;13:2520. doi: 10.1038/s41598-023-29282-9 (PMC9925438; doi:10.1038/s41598-023-29282-9)

Supplementary Information

Tanshinone IIA-loaded nanoparticles and neural stem cell combination therapy improves gut homeostasis and recovery in a pig ischemic stroke model.

Julie H. Jeon[1], Erin E. Kaiser[2,3,4], Elizabeth S. Waters[2,3,4,5], Xueyuan Yang[6], Jeferson M. Lourenco[2], Madison M. Fagan[2,3,4], Kelly M. Scheulin[2,3,4], Sydney E. Sneed[2,3], Soo K. Shin[2,3,7], Holly A. Kinder [2,3,4], Anil Kumar[6], Simon R. Platt[3,8], Jeongyoun Ahn[9], Kylee J. Duberstein[2,3], Michael J. Rothrock Jr.[10], Todd R. Callaway[2], Jin Xie[3,6], Franklin D. West [2,3,4,7], Hea Jin Park[1]*

[1]Department of Nutritional Sciences, University of Georgia, Athens, GA, USA, [2]Department of Animal and Dairy Science, University of Georgia, Athens, GA, USA, [3]Regenerative Bioscience Center, University of Georgia, Athens, GA, USA, [4]Biomedical and Health Sciences Institute, University of Georgia, Athens, GA, USA, [5]Environmental Health Science Department, University of Georgia, Athens, GA, USA, [6]Department of Chemistry, University of Georgia, Athens, GA, USA, [7]Interdisciplinary Toxicology Program, University of Georgia, Athens, GA, USA, [8]Department of Small Animal Medicine and Surgery, University of Georgia, Athens, GA, USA, [9]Department of Statistics, University of Georgia, Athens, GA, USA, [10]US National Poultry Research Center, USDA-ARS, Athens, GA, USA

*Correspondence:

Hea Jin Park, hjpark@uga.edu

Supplementary Figure 1. Original images of Western blot

Figure 2e Occludin

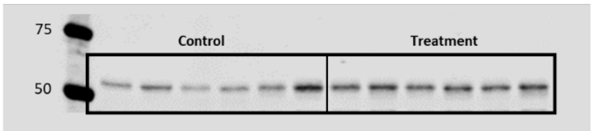

Figure 2e Beta-actin of Occludin

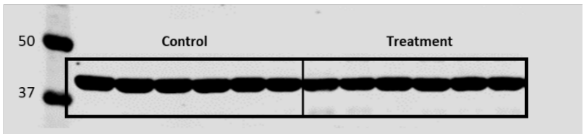

Figure 2e Claudin1

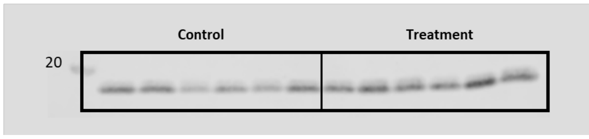

Figure 2e Beta-actin of Claudin1

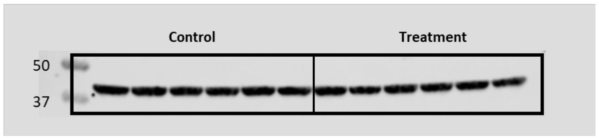

Figure 2e ZO-1

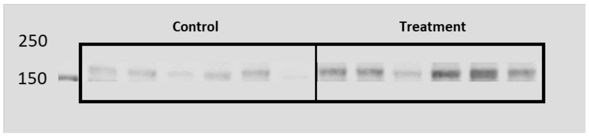

Figure 2e Beta-actin of ZO-1

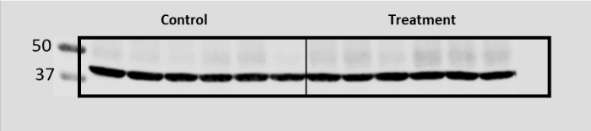

Figure 2b TNFR1

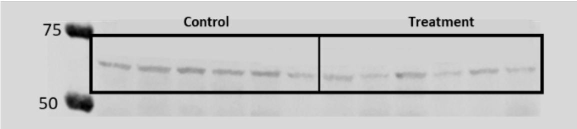

Figure 2b Beta-actin of TNFR1

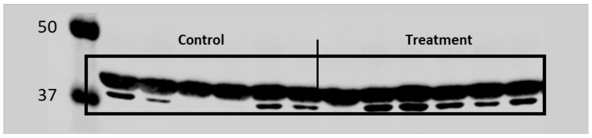

Figure 2c p-Iκbα

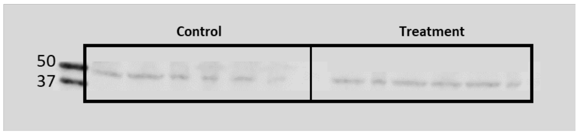

Figure 2c Beta-actin of p-Iκbα

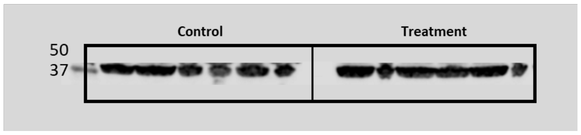

Figure 2c Iκbα

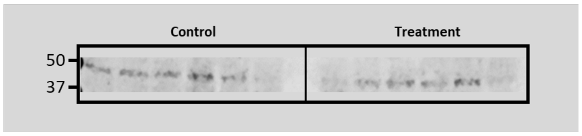

Figure 2c Beta-actin of Iκbα

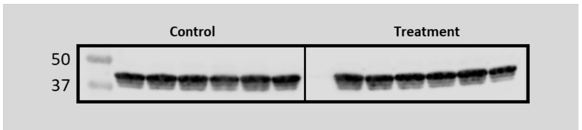

Supplement: Supplementary file 1 — Supplementary Figures. [file 41598_2023_29282_MOESM1_ESM.pdf]
